# Simple and Eco-Friendly Route from Agro-Food Waste to Water Pollutants Removal

**DOI:** 10.3390/ma13235424

**Published:** 2020-11-28

**Authors:** Alena Opálková Šišková, Tomáš Dvorák, Tímea Šimonová Baranyaiová, Erik Šimon, Anita Eckstein Andicsová, Helena Švajdlenková, Andrej Opálek, Peter Krížik, Martin Nosko

**Affiliations:** 1 Institute of Materials and Machine Mechanics, Slovak Academy of Sciences, Dúbravská cesta 9, 84513 Bratislava, Slovakia; tomas.dvorak@savba.sk (T.D.); erik.simon@savba.sk (E.Š.); andrej.opalek@savba.sk (A.O.); peter.krizik@savba.sk (P.K.); martin.nosko@savba.sk (M.N.); 2 Polymer Institute of Slovak Academy of Sciences, Dúbravská cesta 9, 84541 Bratislava, Slovakia; anita.andicsova@savba.sk (A.E.A.); helena.svajdlenkova@savba.sk (H.Š.); 3 Institute of Inorganic Chemistry, Slovak Academy of Sciences, Dúbravská cesta 9, 84536 Bratislava, Slovakia; timea.baranyaiova@savba.sk; 4 Centre for Advanced Materials Application, Slovak Academy of Sciences, Dúbravská cesta 9, 84511 Bratislava, Slovakia

**Keywords:** carbon, agriculture waste, food waste, carrot pulp, sorption, organic pollutants, water purification

## Abstract

The current study reflects the demand to mitigate the environmental issues caused by the waste from the agriculture and food industry. The crops that do not meet the supply chain requirements and waste from their processing are overfilling landfills. The mentioned wastes contain cellulose, which is the most abundant carbon precursor. Therefore, one of the possibilities of returning such waste into the life cycle could be preparing the activated carbon through an eco-friendly and simple route. Herein, the carrot pulp from the waste was used. Techniques such as thermogravimetric analysis (TGA), elemental analysis (EA), scanning electron microscopy (SEM), Fourier-transform infrared spectroscopy (FTIR), Raman spectroscopy, and x-ray diffraction (XRD) were used to investigate the thermal treatment effect during the carbon material preparation. The development of microstructure, phase formation, and chemical composition of prepared material was evaluated. The obtained carbon material was finally tested for water cleaning from a synthetic pollutant such as rhodamine B and phloxine B. An adsorption mechanism was proposed on the base of positron annihilation lifetime spectroscopy (PALS) results and attributed to the responsible interactions. It was shown that a significant carbon sorbent from the organic waste for water purification was obtained.

## 1. Introduction

Nowadays, great attention is paid to carbon-based materials (CM) because of their significant properties, such as lightweight, high strength, low heat, excellent electrical properties, and chemical stability [1]. So far, carbon fibers and particles were applied in various fields of industry, such as building constructions [2], marine [3], electrical conduction, aviation [4], aerospace [5,6], medicine [7,8,9,10,11,12], social protection [13,14], sports accessories [15,16], automotive industry [17,18,19], power engineering [20,21,22], water [23,24] and air [25] treatment.

The most common precursors currently entering carbon materials production are still the petrochemical industry’s everyday products, such as mesophase pitch and polyacrylonitrile (PAN) [1], which exhibit high carbon yield and their advantage is in excellent mechanical and physical properties. However, the current state of production is not sustainable and eco-friendly. Because of the environmental burden, alternative sources are sought for obtaining carbon-based materials [9].

The latest trend in the research and development (R&D) within the world is the full-scale use of renewable sources. Great emphasis is placed on using carbohydrate-based raw materials or proteins; respectively, interest is literally “rising.” Scientists from various parts of the world try to use local natural resources and economically important crops and agricultural and livestock production in given regions. Therefore, a lot of attention is paid to bamboo [26], cotton [27], chitosan [28,29], banana [30] or orange peel [31,32], cactus [33], olive stones [34], coir [35,36], wool [37], hair [38] and animal bones [39,40], silk [41], bacterial cellulose [42], or even manure [43] as well. The advantage of these carbon-based materials is the abundant resource of their precursors. Most of the “natural resources”-derived CM are considered for applications related to (a) energy [26,44,45,46]: supercapacitors for storing electrical or thermal energy, (b) water or air purification: adsorbents [23,24,25], activated carbon [27], and (c) sensors: gas or humidity detection, metal ion presence, or (d) in composites with enhanced functional properties [2]. 

The one group of the pollutants that endanger life in the water but also in the surroundings are synthetic dyes used by textile, cosmetic, pharmaceutical, food, paper, plastic, or printing industries, and they have their advantages in the mentioned sectors; they are cheap and easy to apply. The synthetic dyes have good solubility in water and therefore are harmful to aquatic animals and plants. Thus, it is necessary to eliminate these pollutants from the wastewater [47,48]. Given the increasing problems with waste and environmental pollution, this study’s main idea was to use organic waste from the agricultural or food industry for water purification solution. There was an interest in organic waste, primarily crops that cannot be used due to unsatisfactory quality (do not contain sufficient nutritional value) or the unattractive look redeemed by the sales chains or vegetable pulp from food industry processing, which would eventually end up in landfills or incinerators. In this study, the possibility of fabrication of the carbon materials from natural resources, particularly from the carrot pulp (CP), was verified. Due to its composition (lignocellulose, protein, vitamins, and carotene), a carrot was already considered a potential precursor for carbon-based material fabrication in the past [44,45,46,49]. Besides, intermediates and products after thermal treatment were comprehensively investigated. 

Herein, the conventional heat treatment as an eco-friendly and straightforward route to prepare carbon-based material from CP was used. It consists of a stabilization and carbonization process in the range of laboratory temperature up to 800 °C but without additional chemical treatment to meet environmental aspects. The comprehensive study of chemical structure, physical phases, and carrot pulp morphology after the process’s steps were performed. The prepared carrot pulp-based carbon material was finally tested as an alternative adsorbent for removing synthetic rhodamine B (RhB, cationic nature) and phloxine B (PhB, anionic nature) dyes from the water to verify the efficiency of the prepared carbon material. In the end, the adsorption mechanisms were proposed and attributed to the responsible interactions.

## 2. Materials and Methods

### 2.1. Materials

**Figure 1:**
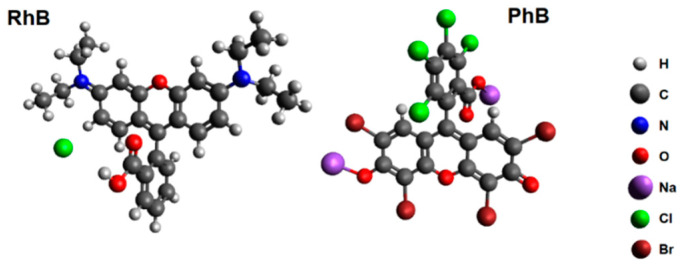
Structural formula of rhodamine B (**left**) and phloxine B (**right**) drawn using the molecule editor Avogadro [50].

The carrots (*Daucus carota subspecies sativus*) were purchased from the commercial food chain. The chloride salt of RhB (≥95%, C.I.45170) and disodium salt of PhB (≥80%, C.I. 45410), used in the adsorption activity testing, were purchased from Sigma-Aldrich (Darmstadt, Germany). The structural formula of RhB cation and PhB anion is shown in Figure 1. Ethanol (>96%) was purchased from Centralchem (Bratislava, Slovakia). Distilled water was prepared by Millipore Milli-Q water system (Merck, Darmstadt, Germany).

#### Preparation of Carbon Material

The 903.25 g of carrot was juiced. The juicer type Vita Juice 2700 W, 1200 rpm, from BOSCH (Gerlingen, Germany), available in a regular household appliance store, was used. The 419.69 g (46.46%) of carrot pulp (CP) was obtained after juicing. Subsequenty, the CP was dried in the conventional laboratory oven (Memmert GmbH, model 100-800, Schwabach, Germany). The temperature was increased every 24 h, from 50 °C up to 250 °C in the oven. Thus, dried and stabilized CP was carbonized from 400 up to 800 °C in a tube electric resistance furnace (Clasic.cz, Řevnice, Czech Republic) under the vacuum of ~10 Pa, with the heating rate of 2 °C·min^−1^, and dwell time of 30 min in the targeted temperature. The cooling was carried out in the oven until room temperature was reached. The samples were taken at temperatures of 100, 150, 200, 250, 400, 600 and 800 °C for subsequent characterization and testing. The samples’ weight losses were measured by laboratory balance with the readability 0.01g (Radwag, Radom, Poland) at ambient temperature 22 °C ± 0.5 °C and at relative humidity 62% ± 0.5%. The experiments were carried on thrice.

### 2.2. Methods

#### 2.2.1. Attenuated Total Reflectance–Fourier Transform Infrared Spectroscopy (ATR-FTIR)

Spectrophotometer Nicolet 8700 (Thermo Fisher Scientific, Madison, WI, USA) was used for ATR-FTIR measurement using thermoelectrically cooled (TEC) fast-recovery deuterated triglycine sulfate (DTGS) detector. Data were acquired in the spectral range 600–4000 cm^−1^ at a resolution of 4 cm^−1^ in the absorbance mode.

#### 2.2.2. Raman Spectroscopy

Raman spectra were excited using the DXR Raman microscope (Thermo Fisher Scientific, Madison, WI, USA) at the 532 nm line of an Nd:YAG laser, with power emission conditions from 10 to 0.1 mW. Peak position was calibrated with a neon glow lamp and a polystyrene standard. Each spectrum was collected in two accumulations of 5 s. Exposure time: 5 s (2 s), numbers of exposures: 2, grating: 900 lines/mm, aperture: 50 μm pinhole (25 μm pinhole), correction for fluorescence, and smooth of all spectra were used. The used power of the laser was 10 mW in the case of 400, 600, and 800 °C, 5 mW for 250 °C, and 2 mW for 100 °C and 200 °C. Raman spectra were analyzed and deconvoluted by the OriginPro 2020 software version 9.7. The G-band and D-band were deconvoluted by Gaussian fitting, and the ratio of intensities was calculated from the bands. 

#### 2.2.3. Elemental Analysis (EA)

Elemental analyzer FLASH 2000 Organic from Thermo Fisher Scientific, Waltham, MA, USA, was used to estimate the C, H, and N content of the CP after each treatment step. The atomic percentage (at.%) of volatile additions was accounted for up to 100%. 

#### 2.2.4. Thermogravimetric Analysis (TGA)

The samples’ thermal stability in the different steps of carbonization was assessed by Mettler-Toledo 851e thermogravimetric analyzer (Columbus, OH, USA), under constant nitrogen flow (50 mL min^−1^) at a heating rate of 10 °C·min^−1^ in the range of 24–800 °C. Approximately 1–3 mg of the sample was weighed and sealed in an aluminum pan. The empty pan was used as a reference.

#### 2.2.5. Scanning Electron Microscopy (SEM)

The morphology of CP in various processing states was observed by scanning electron microscopy (SEM; JSM Jeol 7600F microscope, Tokyo, Japan) equipped with an energy dispersive X-ray detector (EDS) at an accelerated voltage of 10 kV. To avoid charging the samples, they were sputtered with a thin layer of gold. Original JEOL PC_SEM software Ver. 2.1.0.9 (Tokyo, Japan) was used to collect SEM images and process the results.

#### 2.2.6. X-Ray Diffraction (XRD)

X-ray difraction analysis of dried and carbonized samples were performed using diffractometer D8 DISCOVER (Bruker, Karlsruhe, Germany). The device is equipped with an X-ray tube with rotating Cu anode operating at 12 kW. Parallel beam geometry with a parabolic Goebel mirror in the primary beam ws used for all measurements. The X-ray diffraction patterns in grazing incidence set-up with the angle of incidence α = 4° were recorded.

#### 2.2.7. Positron Annihilation Lifetime Spectroscopy (PALS)

The positron lifetime in investigated materials was measured by the conventional fast-fast coincidence spectrometer with a time resolution of 320 ps (FWHM). The device was handmade for laboratory experiments and consists of detectors from SCIONIX (Utrecht, The Netherlands) and electronics from ORTEC (Atlanta, GA, USA). The spectrometer’s resolution function and the correction to the annihilation in the positron source, ^22^Na with activity about 1 MBq, were made using a defect-free pure Al sample with the single positron lifetime 166 ps. Measurements were made at room temperature in air and under vacuum. The measured positron lifetime spectra were analyzed by the LT program [51].

#### 2.2.8. Evaluation of Adsorption Properties of the Carbon Material

Characterization of the adsorption properties of the CP carbonized at 800 °C (carbonized carrot pulp, CCP) was realized by investigating the efficiency of removal of cationic and anionic organic dye, rhodamine B (RhB) and phloxine B (PhB), respectively. 

RhB and PhB aqueous stock solutions were prepared by dissolving in deionized water. The dyes’ concentrations were calculated from Beer–Lambert law, taking into account the molar absorption coefficient of RhB and PhB: ε_543_ nm = 1.06 × 10^5^ mol^−1^·dm^3^·cm^−1^ [52] and ε_552_ nm = 9.87 × 10^4^ mol^−1^·dm^3^·cm^−1^ [53], respectively. By diluting the aqueous stock solutions of RhB and PhB with distilled water in an appropriate ratio, aqueous solutions of RhB and PhB with a dye concentration of 10^−5^ mol·dm^−3^ were prepared.

Prepared solutions were standardized using the method of UV-Vis absorption spectroscopy after appropriate dilution of a small amount of the dye stock solutions in ethanol. The absorption spectra were recorded by UV-Vis absorption spectrophotometer Agilent 8453 (Agilent Technologies, Waldbronn, Germany) over the entire wavelength range (190–1100 nm) in quartz cuvette of path length 10 mm.

Adsorption experiments were realized by adding the necessary amount of CCP to a dye solution (10^−5^ mol·dm^−3^) with a volume of 70 mL. The carrot pulp carbonized at 800 °C was crushed to a fine powder in a mortar just before the experiments. In the case of both dyes, two systems that differ in the ratio of the amount of dye to the mass of CCP sample were prepared (Table 1). The CCP sample was added to the dye stock solution while stirring.

The reaction mixtures were stirred continuously at laboratory conditions for 5 days. After certain time intervals from adding the carbon sample to the dye solution (15 min, 30 min, 45 min, 60 min, 90 min, 120 min, 150 min, 180 min, 210 min, 240 min, 270 min, 5 h, 6 h, 7 h, 8 h, 9 h, 10 h, 22 h, 24 h, 26 h, 28 h, 30 h, 48 h, 52 h, 54 h, 5 days), ~ 2 mL of the reaction mixture was taken and centrifuged at 12,000 rpm for 15 min. After the separation of the carbon particles with adsorbed dye molecules, the concentration of free RhB cations (respectively, of PhB anions) in the supernatant was determined from the dye’s measured absorbance value at the wavelength of its maximum absorption. The concentration of non-adsorbed dye molecules at respective reaction times was calculated from Beer–Lambert law, using the determined values of the molar absorption coefficient of RhB and PhB in water: ε_554_ nm = 6.02 × 10^4^ mol^−1^·dm^3^·cm^−1^ and ε_539_ nm = 5.10 × 10^4^ mol^−1^·dm^3^·cm^−1^, respectively.

The removal efficiency of the carbonized pulp sample at the respective reaction time (*r_t_* (%)) was calculated using Equation (1):

(1) $$r_{t}\left(\%\right)=\frac{c_{0}\left(\mathrm{dye}\right)-c_{t}\left(\mathrm{dye}\right)}{c_{0}\left(\mathrm{dye}\right)}\times 100$$

Symbols *c*_0_ (dye) and *c_t_* (dye) represent the initial concentration of the dye (10^−5^ mol·L^−1^) and the concentration of non-adsorbed dye molecules in the supernatant separated from the reaction mixtures after different reaction times (*t*).

## 3. Results

### 3.1. Preparation of the Carbon-Based Material from the Carrot Pulp

**Figure 2:**
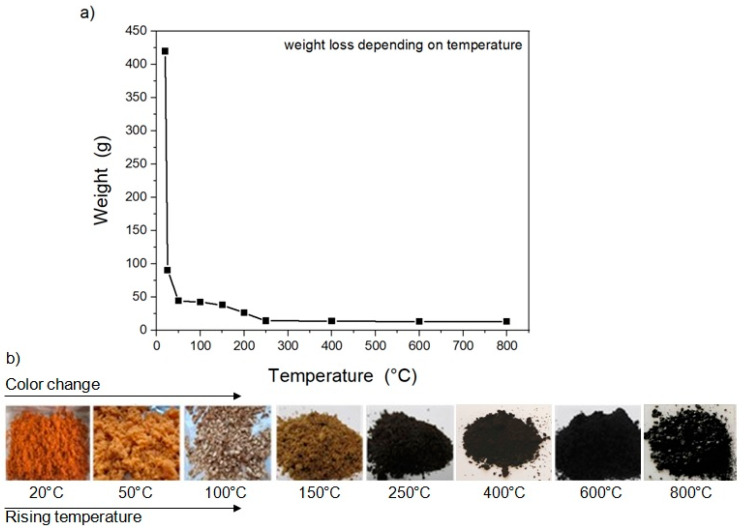
Effect of weight loss (**a**) and color change (**b**) during the stabilization and carbonization processes of the carrot pulp.

Carrot pulp is losing weight with increasing temperature during the thermal stabilization and carbonization. The trend of weight decrease is shown in Figure 2a. The color changes indicated the increase in the carbon content. The color changes depending on the increasing temperatures are shown in Figure 2b.

### 3.2. Characterization of Investigated Material

#### 3.2.1. Thermal Analysis

**Figure 3:**
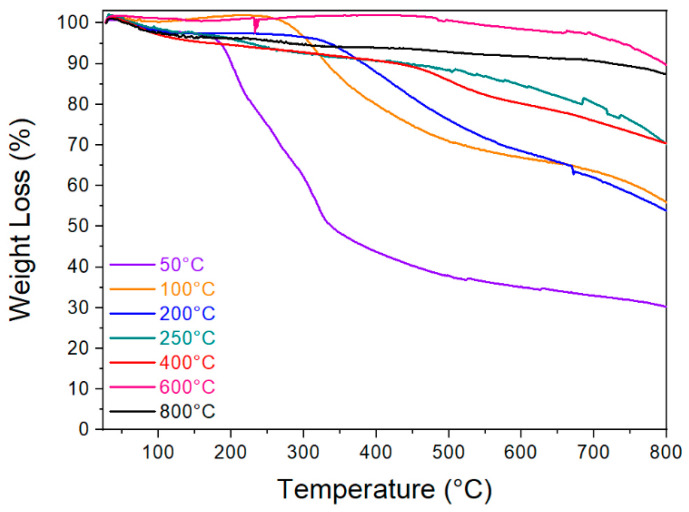
Thermogravimetric analysis of carrot pulp stabilized and carbonized at temperatures between 50 and 800 °C.

The TGA results of the investigated material are shown in Figure 3. The results indicate an increased thermal stability of the CP with increasing treatment temperature.

The mass yields resulting from the thermogravimetric analysis are summarized in Table 2. 

#### 3.2.2. Raman Spectroscopy Analysis

**Figure 4:**
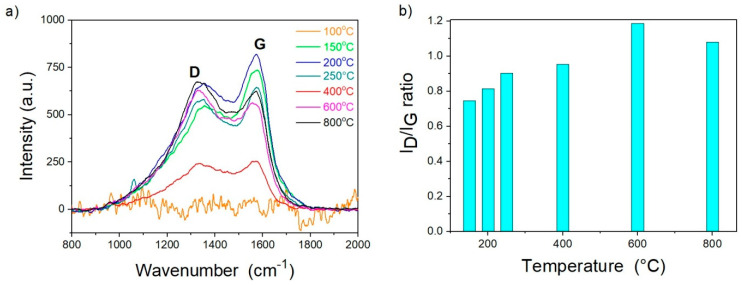
(**a**) Effect of the temperature on the Raman spectra of carrot pulp (CP) treated at various temperatures. (**b**) I_D_/I_G_ ratio of the pulps on temperature.

The Raman spectra of the carrot pulp in dependence on the temperature of stabilization and carbonization are shown in Figure 4. The Raman spectra confirmed the formation of the stable C–C bonds, indicating D- and G-bands’ formation.

The Raman spectra consist of two prominent bands observed at 1580 and 1340 cm^−1^. The peak at 1580 cm^−1^ (G-band) is attributed to the vibration of sp^2^ hybridized carbon atoms in a 2D hexagonal lattice (order graphitic carbon features). The peak at approx. 1340 cm^−1^ (D-band) is associated with the vibrations of carbon atoms with dangling bonds in-plane terminations of the disordered graphite from the defects and disorders of structures in carbon materials [54,55].

The D-band intensity ratio to G-band (I_D_/I_G_) peaks depending on the type of graphitic materials and reflecting the graphitization degree are summarized in Table 3.

#### 3.2.3. Elemental Analysis

The main elemental composition of the surface of carrot pulp after each stage of heat treatment is shown in Table 4. The carbon (C) content in the sample is increasing significantly; oxygen (O) and hydrogen (H) is decreasing with increasing temperature. The nitrogen content does not change significantly during heat treatment.

#### 3.2.4. FTIR Analysis

**Figure 5:**
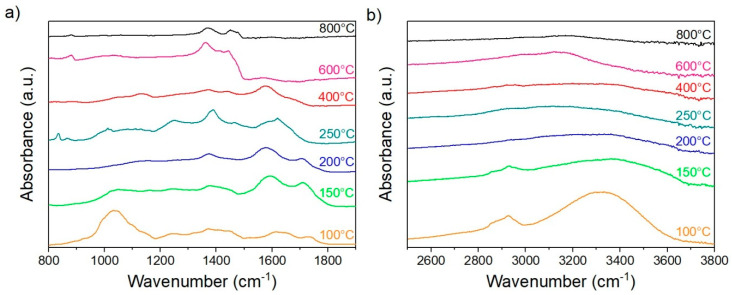
Fourier-transform infrared (FTIR) spectra of CP were processed at different temperatures in the range of wavenumber of 800–1900 cm^−1^ (**a**) and 2500–3800 cm^−1^ (**b**).

Samples were taken to be analyzed by FTIR spectroscopy. The reason is to observe the changes in the chemical groups present in the investigated material and compare the samples’ chemical development before heat treatment and after all stages of the treatment from 100 °C up to 800 °C. The results are compared in Figure 3. The obtained spectra are divided into two groups according to the wavenumber from 800 cm^−1^ up to 1900 cm^−1^ (Figure 5a) and from 2500 cm^−1^ up to 3800 cm^−1^ (Figure 5b).

Table 5 shows the significant bands assigned to the corresponding chemical groups.

#### 3.2.5. Morphological Analysis

**Figure 6:**
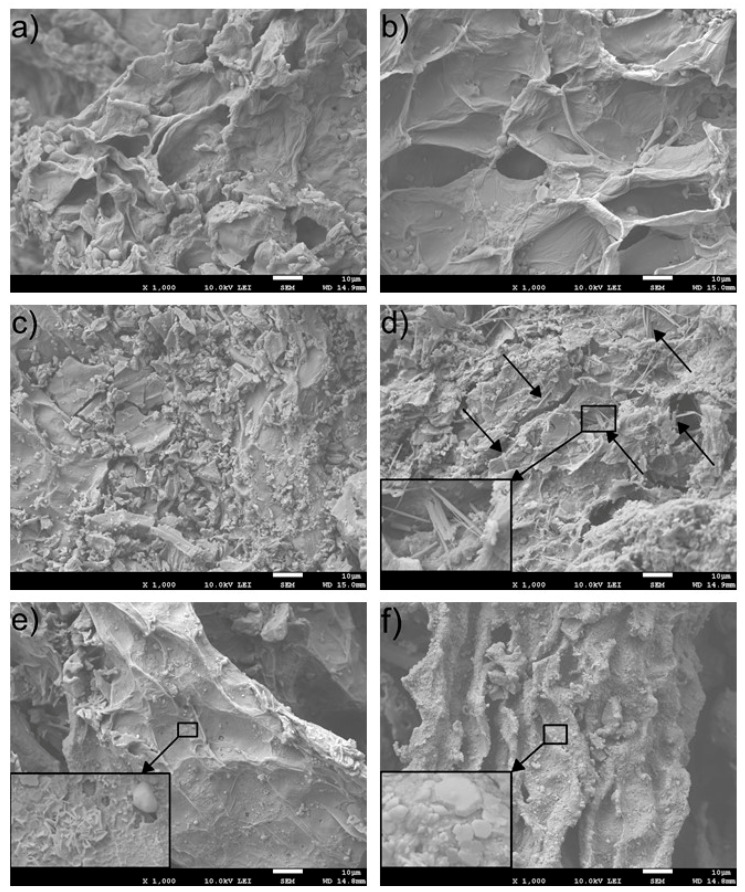
Scanning electron microscopy (SEM) microstructure of the heat-treated carrot in dependence on the temperature (**a**) 150, (**b**) 200, (**c**) 250, (**d**) 400, (**e**) 600, and (**f**) 800 °C.

**Figure 7:**
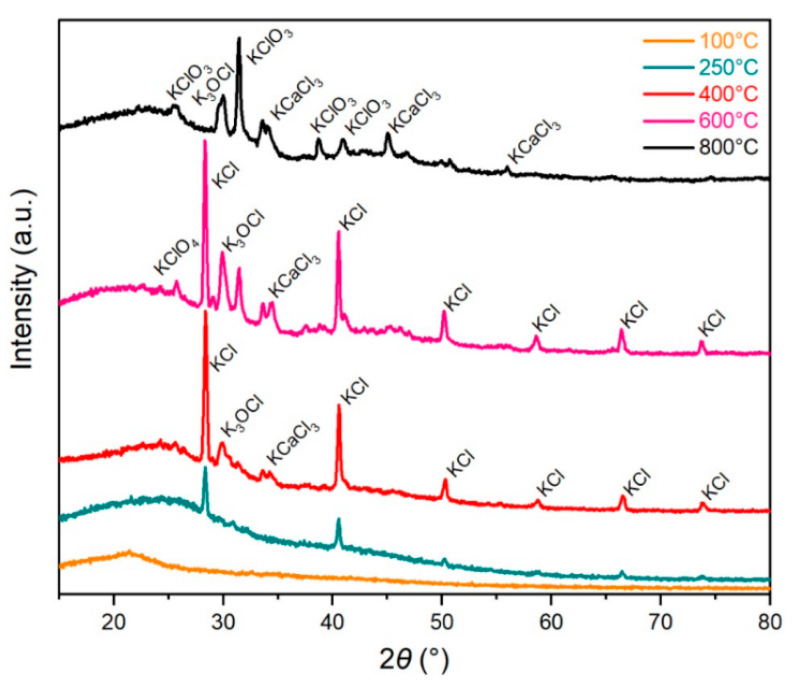
X-ray diffraction (XRD) pattern of the heat-treated carrot in dependence on the heat treatment temperature.

Through SEM, the morphology of the carrot pulp and its cell walls was observed. The morphology was changing significantly with the increase in the temperature. The physical phases of some compounds are formed, and some were also disappearing with the increasing temperature that was observed through the “needle-like” (Figure 6d) and “rose-like” (Figure 6e) objects on the samples between 400 and 800 °C. XRD confirms the physical phase’s changes on the cell walls (Section 3.2.6 and Figure 7). The morphology is shown in Figure 6.

#### 3.2.6. X-Ray Diffraction Analysis

Formation and extinction of physical phases during the thermal treatment were investigated by X-ray diffraction analysis. There are phases assigned to the compounds containing in the samples in Figure 7. The presence of “needle-like” and “rose-like” objects was confirmed by morphology investigation at carbonization temperatures (Section 3.2.5 and Figure 6). Some of the items were disappearing with the increasing temperatures.

### 3.3. Organic Pollutants Removal from the Water

To characterize the selective dye removal performances of the present obtained bio-based CCP adsorbents, the CCP was investigated concerning their uptake of removing dyes phloxine B (PhB) and rhodamine B (RhB) models. 

**Figure 8:**
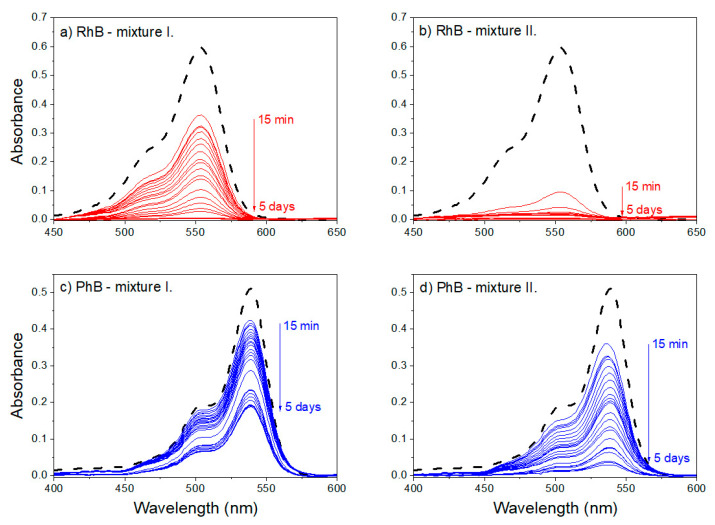
Absorption spectra of the supernatant obtained by centrifuging the mixture of dye and CCP sample with the ratio of the amount of RhB to the mass of CCP sample of 1.60 µmol·g^−1^ (**a**) and 0.40 µmol·g^−1^ (**b**) and of PhB to the mass of CCP sample of 1.60 µmol·g^−1^ (**c**) and 0.40 µmol·g^−1^ (**d**) (all solid lines); absorption spectra of the RhB (**a**,**b**) and PhB (**c**,**d**) in water are displayed by dashed lines.

The absorption spectrum of PhB and RhB in water (dashed lines) and spectra of the supernatant obtained by centrifuging the mixture of dye and CCP sample after various reaction times (solid lines) is shown in Figure 8. Figure 8a,b correspond to the supernatant obtained from the mixture of RhB and CCP sample, with the ratio of the amount of RhB to the mass of CCP sample of 1.60 and 0.40 µmol·g^−1^ (RhB—mixture I. and RhB—mixture II., respectively). Figure 8c,d correspond to the supernatant obtained from the mixture of PhB and CCP sample, with the ratio of the amount of PhB to the mass of a CCP sample of 1.60 and 0.40 µmol·g^−1^ (PhB—mixture I. and PhB—mixture II., respectively). Red and blue arrows indicate the direction in which the reaction time increases in the range from 15 min to 5 days.

**Figure 9:**
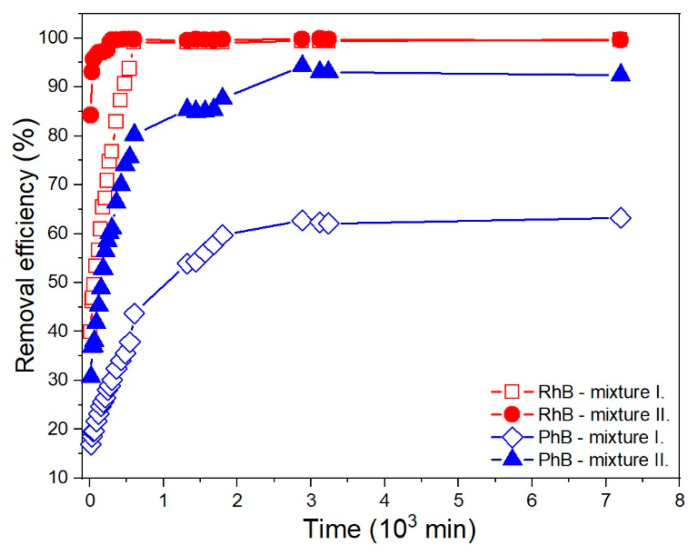
Evolution of RhB and PhB removal efficiency with time for the mixtures with the ratio of the amount of dye to the mass of CCP sample of 1.60 µmol·g^−1^ (mixture I.) and 0.40 µmol·g^−1^ (mixture II.).

The adsorption properties of CCP toward PhB and RhB are shown in Figure 9. 

#### Free Volume Analysis

Positron annihilation spectroscopy offers a very sensitive probe to the determination of investigated material free volume [56]. Free volume analysis was carried out by PALS to obtain information about the present size and the possible adsorption mechanism of the synthetic dye into the CCP from the water. For the free volume analysis, the RhB dye was used. The radii of microporous free-volumes of all investigated materials CCP carbonized at 800 °C, RhB dye in bulk, and the CCP after adsorption of RhB from water (CCP/RhB) were calculated from the lifetime *t*_2_ according to Equation (2).

(2) $$t_{2}=0.260\times 1-\left(\frac{R}{R+3.823}\right)+\frac{1}{2}\pi \times \frac{\sin2\pi R}{{\left(R+3.823\right)}^{-1}}$$

*R* is the spherical hole radius, and *t*_2_ is the second lifetime component. The results are in Table 6.

**Figure 10:**
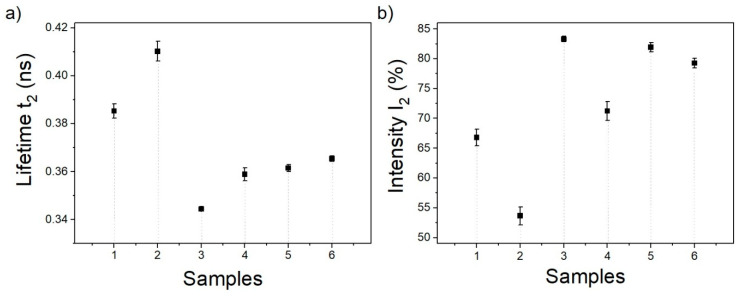
Lifetime t_2_ (**a**) and the relative intensity I_2_ (**b**) for the CCP, rhodamine B, and their composite. 1—CCP measured in air, 2—CCP measured in vacuum, 3—RhB measured in air, 4—RhB measured in vacuum, 5—CCP/RhB measured in air, 6—CCP/RhB measured in vacuum.

The lifetime *t*_2_ and intensities *I*_2_ provided the information about the internal structure of the investigated materials. *T*_2_ in the hundreds of picoseconds and intensity in % are summarized for all three types of materials studied in air and vacuum in Figure 10.

**Figure 11:**
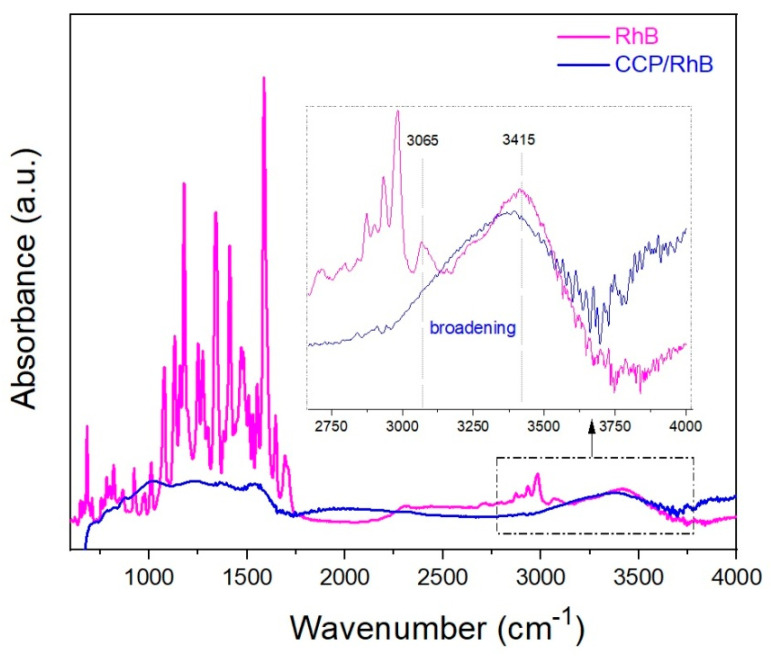
FTIR results of RhB (magenta line) and the “composite” material CCP/RhB (blue line) in the range 600–4000 cm^−1^.

Figure 11 shows the FTIR results of RhB and the “composite” material CCP/RhB. The broadening of the peak with the maximum 3415 cm^−1^ and suppressing the peaks in the range of the wavenumbers 3065 and 3253 cm^−1^ pointed to the possible interactions in the CCP/RhB system.

## 4. Discussion

Bio-based carbon material from the carrot pulp was prepared by thermal treatment without any chemical additives. Both intermediates and products were comprehensively studied after each level of the thermal treatment. An exponential decrease in the weight of CP, due to the evaporation of the chemically and physically bound water or gas fleeing from the surface, [57] is seen in Figure 2. The crude fiber in carrot roots consists of 71.7%, 13.0%, and 15.2% cellulose, hemicellulose, and lignin, respectively [6,58]; therefore, only a slight decrease was observed during the subsequent temperature increase above 250 °C. It could be explained by hemicellulose and lignin residues decomposition [57]. Moreover, weight loss is accompanied with color change, as documented in Figure 2. The orange color of freshly juiced CP is getting darker by increasing temperature related to the increasing content of C–C bonds supported by Raman and FTIR as shown in Figure 4 and Figure 5. The G- and D-band formed during stabilization at 150 °C. Above 200 °C, black color is observed, which suggests the onset of the carbonization.

Figure 3 shows the thermal stability of CP after thermal treatment at different stabilization and carbonization temperatures up to 800 °C. Typical TGA curve as observed for the cellulosic materials contains hemicellulose and lignin [59]. The samples stabilized at 100 °C and 200 °C exhibited a significant weight loss as the cellulose continually degraded. The mass yields are listed in Table 2. All samples were carbonized at a temperature above 400 °C, and gains between 60 and 80% were observed up to 800 °C. The outcomes indicate that the increase in carbonization temperature results in higher thermal stabilities. The critical temperature is set at 250 °C when more than 50% of mass yield is characteristic.

The Raman spectra of carrot pulp in dependence on temperature are shown in Figure 4. As expected, two peaks at approx. 1580 cm^−1^ (1557–1596 cm^−1^) and 1340 cm^−1^ (1326–1355 cm^−1^) are observed for each temperature over 100 °C. The intensity ratio of these D-band to G-band (ID/IG) peaks depends on the type of graphitic materials. It reflects the graphitization degree, summarized in Table 3. The I_D_/I_G_ ratio was low at 400 °C and below, indicating more graphitic carbon than non-crystalline carbon within CP. During the carbonization, above 600 °C, the I_D_/I_G_ ratio increased to 1.2 (the highest value observed in this study). Afterwards, with the treatment temperature increase to 800 °C, the I_D_/I_G_ ratio decreased.

The above-mentioned indicates that the carbon materials’ graphitic degree decreases with increasing processing temperature, which is not unique for carbon materials and puts the achievements in good agreement with the available literature [60,61,62]. The D-band presence also indicates disorders or defects in CCP that could be present in the form of pores.

The main elemental composition of CP surface is shown in Table 4. As seen in the table, C is the most abundant constituent of CP, with increasing carbonization temperature, the ratio of C and N increase while O and H decrease. The N ratio in the carbon material varies slightly from the temperature dependence. The C amounts above 75% at 800 °C. This behavior during the drying and carbonization process was already reported in the literature [63]. From EDS analysis, other minor elements such as Na, Mg, Cl, K, and Ca present within the cell walls have been observed as published in the literature [64]. This observation agrees with previously reported data in many studies related to food chemistry [58].

Figure 5 shows the CP FTIR spectra treated under different stabilization and carbonization temperatures from 100 up to 800 °C. The analyzed spectra agree with the literature, exhibiting typical carbohydrate spectra [65]. The studied spectra are divided into two regions. The first region, the so-called “fingerprint “region, which could be seen between 800 and 1800 cm^−1^, is shown in Figure 5a. In this case, the results are remarkably similar to the FTIR spectra of wood, as described in Table 5. Wavenumbers mentioned in the table are present in the spectra more or less up to 400 °C. The peaks decreasing intensity even until disappearing the bonds in the region between 1000 and 1100 cm^−1^ are related to the fact that hemicellulose decomposes at 150–350 °C, cellulose at 270–350 °C. Lignin is the most stable and decomposes at 250–500 °C, hence the band 1235 cm^−1^ related to syringyl rings of lignin disappears above 400 °C [66]. The spectrum of the sample treated at 600 °C revealed that only the bonds at 1372 and 1424 cm^−1^ remained present, which correspond to C–H deformation and aromatic skeletal vibration combined with C–H in-plane deformation. Second is the region of the –OH stretching in the wavenumber 3000–3700 cm^−1^ and –CH_3_, –CH_2_ stretching between 2700 and 2900 cm^−1^, as it is shown in Figure 5b. These broad peaks disappear with increasing temperature. The most significant decrease in peaks is observed at a temperature of 150 °C, which complies with the Raman results. The decrease or even absence of the described region’s peak suggests that the sample is mainly getting rid of moisture. The presence of carbon is increasing by rising temperature. Mentioned peaks in the spectrum of CP treated above 600 °C almost wholly disappeared. This behavior is common in the carbonized samples from natural resources. 

The effect of drying was observed on the carrot structure by SEM, as shown in Figure 6. In general, the carrot microstructure after heat treatment consists of the crushed cells (both ground and vascular [67,68]) due to juicing the carrot before drying and heat treatment. No walls tearing, macropores, or a sponge effect as in case of freezing treatment [69], microwave [70], or ultrasonic radiation [71] were observed, nor was the presence of voids (small cavities). However, the cell walls character is affected by the treatment temperature; the walls are corrugated up to 250 °C and have a somewhat brittle nature above this temperature, probably due to the decreasing amount of O and H, as reported in Table 4. Moreover, there is a “needle-like” phase observed in the microstructure at a temperature of 400 °C, while “rose-like” structures are revealed at a temperature of 600 °C. At 800 °C, the cell walls are less opened than those treated at I_D_/I_G_ lower temperature, which indicates enhanced tissue shrinkage. Cell walls treated at 800 °C are entirely covered by the blocky, like the phases depicted in Figure 6.

Different phases in samples after carbonization at different heat treatment temperatures are shown in Figure 7. There is a primary growth of KCl above 250 °C up to the temperature 600 °C, while KCl is not detected at 800 °C. Moreover, K_3_OCl and KCaCl_3_ are identified at 400 °C, and 600 °C also presented KClO_4_. At 800 °C, the predominant phase is KClO_3_. Within the range 20–30° (2*θ*), a low-intensity signal is observed, which indicates amorphous carbon for all treatment temperatures [72,73]. The undeveloped character of carbon products is in agreement with the results of Raman spectroscopy (Figure 4). N via EDS and elemental analysis has been qualitatively detected but did not detect any phases containing N via XRD.

The XRD pattern of the sample dried at 100 °C indicates the typical amorphous structure, confirmed by the broad peak appearing at approximately 2*θ* = 22°. Besides organic components, carrot contains many metal elements naturally as K, Na, Mg, and Ca in a different form [64]. The decomposition of the organic substances and organic substances containing metal elements occurs during heat treatment. As mentioned above, the XRD pattern of initially stabilized and carbonized carrot pulp, except the sample carbonized at 800 °C, consists of the diffraction of KCl as a primary inorganic salt. The presence of the KCaCl_3_ at 400 and 600 °C could be explained as the reaction of KCl and CaCl_2_. KCl and CaCl_2_ can create a eutectic mixture, which could occur around 600 °C in the melted phase [74]. Therefore, the formation of different morphologies of salts on the carbon surface (Figure 6) as crystallization products from melt phases was considered.

It was observed that, during the carbonization of the different natural carbon material (as the example: mesophase pitch, charcoal) with a high content of a potassium salt, the surface salt complexes are created on the carbon surface [75,76]. They are observed at high temperatures, 700–900 °C, and are responsible for the catalytical gasification of carbon. The mentioned surface layer shows high reactivity. It has been supposed that the presence K_3_OCl, KClO_4_, KClO_3_ are product surface salt complexes and KCl. Concerning substances are stable at low temperatures in an environment of carbon (KClO_4_ below 520 °C and KClO_3_ below 320 °C) [77,78], and therefore the formation K_3_OCl, KClO_4_, KClO_3_ was expected during the cooling of samples. 

After the addition of the CP sample to a dye (RhB and PhB, respectively) solution, and the subsequent separation of the supernatant after various reaction times, a gradual decrease in the intensity of the main absorption band was observed in the case of both dyes. The shape of the main absorption band in the visible (Vis) range remained unchanged in the case of all the investigated systems (Figure 8, solid lines). Changes in the shape of the absorption spectra of the selected dye molecules were not occur even in the UV part of the spectra (not shown). The observed decrease in the intensity of the main absorption band of RhB and PhB indicates that the addition of the CP sample to water solutions of organic dyes is resulted in the adsorption of dye molecules at the surface of the carbonized sample.

With increasing reaction time, an increase in the removal efficiency of the CCP sample (*r_t_* (%), Equation (1)) was observed. The most intense increase in the calculated values of the removal efficiency occurred during shorter reaction times, while a significant effect of the *n* (dye)/*m* (CCP) ratio on the initial value of the removal efficiency was observed: initial removal efficiency (*r_i_* (%)) is higher for the reaction mixtures with a lower ratio of the amount of dye to the mass of CP sample. An increase in the mass concentration of the CCP sample led to the reaching of the maximum value of removal efficiency (*r_e_* (%)) in shorter reaction time in the case of both investigated dyes (Figure 9). 

For the reaction mixtures of CCP with RhB, the removal efficiency of the CCP sample after 15 min of reaction time was *r_i_* (%) = 39.8% and 84.2% for the mixtures with an *n* (RhB)/*m* (CCP) ratio of 1.60 and 0.40 µmol·g^−1^, respectively. The comparison of the obtained value of the removal efficiency after the reaching of the equilibrium state (*r_e_* (%) = 99.4% and 99.6%) indicates that complete removal of RhB molecules is achieved even in the case of the reaction system with a higher *n* (RhB)/*m* (CCP) ratio. Achieving the equilibrium state in the systems with a higher and lower *n* (RhB)/*m* (CCP) ratio required 10 and 5 h, respectively (Figure 8a,b and Figure 9). 

In contrast to reaction mixtures with rhodamine cations, the addition of the prepared CCP sample to a solution of PhB did not lead to the complete removal of the dye anions from the mixture (Figure 8c,d). After 15 min of reaction time, the initial value of the removal efficiency was *r_i_* (%) = 16.9% and 30.7% (for the systems with *n* (PhB)/*m* (CCP) ratio of 1.60 µmol·g^−1^ and 0.40 µmol·g^−1^, respectively). The presence of non-adsorbed PhB anions in a low concentration after reaching the maximum removal efficiency (*c*_e_ (PhB) ≈ 5 × 10^−7^ mol·dm^−3^) was observed even in the supernatant obtained from the mixture with a lower *n* (PhB)/*m* (CCP) ratio, which corresponds to a value of *r_e_* (%) = 94.3%. The maximum removal efficiency of the CCP sample for the system with the ratio *n* (PhB)/*m* (CCP) = 1.60 µmol·g^−1^ was *r_e_* (%) = 63.3%. The equilibrium state characterized by a maximum value of removal efficiency was reached after 52 and 48 h (for the systems with higher and lower *n* (PhB)/*m* (CCP) ratio). 

Free volume analysis by PALS provided important information about the properties of the CCP and the system CCP/RhB obtained from the dye adsorption experiments. Generally, positron annihilation occurs from different states and sites (bulk, defects, large cavities), manifested by the different mean lifetime that characterize these states. The lifetime of positrons in a substance depends on its electron density. Lifetime provides information about the internal structure of the investigated material. 

In this particular case, the formation of positronium (Ps) (bound state of the electron with positron), characterized by a long lifetime in the ns range, was not found in any investigated materials. The short component (*t*_1_) is usually attributed to the annihilation of the positron in bulk; the second (*t*_2_), longer component, is attributed to the annihilation in a place with low electron density, for example, in different types of defects and small cavities or the annihilation of surface-trapped positrons [78]. It is known that Ps is not formed in pure carbon in different forms [79,80,81] or it creates in exceptional cases only [82], so it is impossible to analyze any mesopores in the material using standard PALS porosimetry based on the measured ortho-Ps lifetime and an appropriate models [83,84]. However, a short component associated with defect states is useful for characterizing micro-pores and defects that are not detectable by standard methods, such as BET (Brunauer, Emmett and Teller). Likewise, RhB also belongs to the group of substances with suppressed Ps formation, as follows from these measurements. For such materials, without the creation of Ps, the formula for the estimation of pore size from the second component of lifetime *t*_2_ spectra could be used according to the work of Liao et al. [85]. The presence of a significant number of open volume defects detected by PALS in the CCP resulted from the *t*_2_ spectra (410 ps, 54%). It was also confirmed by Raman spectroscopy. The presence of D-band (1340 cm^−1^) was a consequence of disorders and defects in carbon material. The radii of microporous free volumes for all investigated materials were calculated from the lifetime *t*_2_ according to Equation (2). The positron lifetime *t*_2_ (Figure 10a), intensities (*I*_2_) (Figure 10b), both for the investigated material measured in vacuum and the estimated radius of micropores of CCP, are summarized in the Table 6. The table did not include values for material monitored in air because of lifetime *t*_2_ in CCP is affected by the parameters of surroundings, and the significant shift of *t*_2_ values measured in the air indicates the effect of the oxygen and humidity in open spaces [56,86]. The lifetime *t*_2_ and their relative intensities *I*_2_ of the composite CCP/RhB show the minimal influence of oxygen from the air on the spectral parameters. Both the *t*_2_ and the *I*_2_ are similar to those with pure RhB under vacuum. These findings suggest that annihilation in the carbon matrix is minimal, and positrons annihilate predominantly in RhB, on the carbon matrix surface. 

FTIR analysis of RhB and system CCP/RhB revealed the peak’s broadening with the maximum at approx. 3415 cm^−1^, which corresponds to oxygen-containing functional groups accessible for RhB with abundant adsorption sites (Figure 11). This indicates the formation of H-bonds in the CCP/RhB system [43,87]. A weak intensity band at 3065 cm^−1^ belongs to aromatic C–H vibrations, which also disappeared after adsorption RhB into the CCP [88]. This disappearing of the absorption peak could indicate the π-π interaction between the RhB and CCP. The electrostatic interactions could be responsible for selectivity in the dye’s adsorption, and this could be the explanation of the different adsorption efficiency of cationic (RhB) and anionic (PhB) dye from the solutions (99.4% and 63.3%, respectively) with the higher *n* (dye)/*m* (CCP) ratio (Section 3.3). The observation could be concluded that several interactions contribute to the adsorption mechanism, which is in good agreement with the already published studies [43,89,90].

As presented above, this study offers an alternative carbon adsorbent preparation from food waste or agricultural waste by a simple ecological route to clean polluted water. In the future, the research will be focused on the higher yield of carbon and the ability of CCP to clean water from heavy metals and medicaments or their metabolites. Likewise, our team will also cooperate with the sewage treatment plant to use the large-scale carbonized municipal bio-based waste. Other applications of such carbon material on the base of agro-food waste are being sought.

## 5. Conclusions

This study’s main idea was to eliminate the environmental burden by returning the organic waste from the agro-food industry into the life cycle and using the product for a water purification solution. The intermediates and products after each level of thermal treatment were comprehensively investigated as well. 

The results of the study can be summarized as follows:

- The eco-friendly and straightforward process can be used to fabricate carbon sorbent to purify wastewater with significant efficiency.
- Thermal treatment leads to the production the carbon-based material with a maximum carbon content of 75.5% from an alternative natural resource without using additional chemicals.
- There is a significant change of the cell wall morphology with treatment temperature in the phase’s development phase. Furthermore, SEM and XRD analysis reveal different inorganic salts on the surface of carbonized carrot pulp, which could be products of molten salt of eutectic KCl–CaCl_2_ products of surface organic-potassium salt complexes.
- The CP treated at a temperature of 800 °C (CCP) could adsorb the organic dye (RhB and PhB). The removal efficiency of the CCP sample increased by increasing time independently of the dye type. The removal efficiency of the cationic type of dye is more than 99%, and the anionic kind of dye is more than 94% into the CP-based carbon material.

## Figures and Tables

**Table 1:** Composition of the mixtures of organic dye (rhodamine B or phloxine B) with carbon sample. The volume of the mixtures was always 70 mL. Symbols *c* (dye) and *c*_m_ (CCP) denote a concentration of rhodamine B (respectively, phloxine B) and mass concentration of the pulp sample. Symbols *n* (dye)/*m* (CCP) corresponds to a dye ratio to the mass of the pulp sample.

Mixture	*c* (dye) (mol·L^−1^)	*c*_m_ (CCP) (g·L^−1^)	*n* (dye)/*m* (CCP) (µmol·g^−1^)
I.	10^−5^	6.25	1.60
II.	10^−5^	25	0.40

**Table 2:** Mass yields of carrot pulp after thermal treatment at different temperatures, 50 up to 800 °C. The mass yield was obtained from the values provided by thermogravimetric analysis (TGA) software.

Treatment Temp. (°C)	50	100	200	250	400	600	800
**Mass Yield (%)**	22.37	43.67	44.14	53.67	63.15	76.20	80.37

**Table 3:** Intensity ratios of D-band to G-band (I_D_/I_G_) and Raman shifts of the samples’ bands peaks.

Temperature (°C)	I_D_	λ_D_ (cm^−1^)	I_G_	λ_G_ (cm^−1^)	I_D_/I_G_
100	–	–	–	–	–
150	547.8	1355	735.5	1576	0.74
200	666.6	1351	818.2	1575	0.81
250	580.5	1353	643.5	1576	0.90
400	241.5	1337	253.2	1571	0.95
600	628.6	1326	561.9	1557	1.12
800	672.5	1333	622.9	1573	1.09

**Table 4:** Elemental analysis of organic elements C, N, O, and H of CP after different stabilization and carbonization temperature in at.%.

Temperature (°C)	C	N	O	H
100	42.0	0.8	51.5	5.7
150	51.5	1.5	43.1	3.9
200	54.2	2.0	40.6	3.2
250	55.0	2.1	41.0	1.9
400	66.2	2.4	28.5	2.9
600	68.6	2.3	27.6	1.5
800	75.5	1.9	21.8	0.8

**Table 5:** Table of the present bonds and corresponding wavenumbers in the CP.

Wavenumber (cm^−^^1^)	Bands
**3000–3700**	–OH Stretching
**2700–2900**	–CH_3_, –CH_2_ Stretching
**1732**	C=O in Hemicellulose
**1649**	C–O–H Absorbed and C–O Conjugated
**1543**	Aromatic Skeletal Vibration C=O Stretch
**1460**	C–H Deformation, Asymmetric at –CH_3_ and –CH_2_
**1424**	Aromatic Skeletal Vibration Combined with C–H in-Plane Deformation
**1372**	C–H Deformation
**1235**	Syringyl Rings and C= in Lignin
**1109**	–OH Activation in Cellulose and Hemicellulose
**1076**	C–H, C–O Deformation
**1056**	C–O Stretching in Cellulose and Lignin
**1031**	Aromatic C–H in-Plane Deformation, C–O Deformation, and Primary Alcohol
**998**	C–O–H Stretching in Cellulose and Hemicellulose

**Table 6:** Positron lifetime *t*_2_, their relative intensity *I*_2_ (at vacuum), and the estimated radius of micropores for CCP, rhodamine (RhB), and their composite.

Researched Material	t_2_ (ns)	I_2_ (%)	R (Å)
**CCP**	0.410 ± 0.004	54 ± 2	2.9
**RhB**	0.359 ± 0.003	71 ± 2	2.4
**CCP/RhB**	0.365 ± 0.002	79 ± 1	2.5
